# Heart Failure with Recovered Ejection Fraction in Patients with Vinculin Loss-of-function Variants

**DOI:** 10.1007/s12265-023-10421-6

**Published:** 2023-08-07

**Authors:** Laura Zahavich, Rajadurai Akilen, Kristen George, Seema Mital

**Affiliations:** 1 Department of Genetic Counselling, Hospital for Sick Children, University of Toronto, Toronto, Ontario Canada; 2 Department of Molecular Genetics, University of Toronto, Toronto, Ontario Canada; 3 Labatt Family Heart Centre, Hospital for Sick Children, Toronto, Ontario Canada; 4 Genetics and Genome Biology Program, Hospital for Sick Children, Toronto, Ontario Canada; 5 Department of Pediatrics, Hospital for Sick Children, University of Toronto, 555 University Avenue, Toronto, ON M5G 1X8 Canada; 6 Ted Rogers Centre for Heart Research, Toronto, Ontario Canada

**Keywords:** Dilated cardiomyopathy, Vinculin, Myocardial recovery, Heart failure, HFrEF, HFrecEF

## Abstract

Predictors of myocardial recovery in heart failure (HF) are poorly understood. We explored if vinculin (*VCL*) variants are associated with myocardial recovery in dilated cardiomyopathy (DCM). Six infants with DCM with a *VCL* loss-of-function (LOF) variant were identified. Median age at diagnosis was 2 months, median LV ejection fraction was 24%, and median LV end-diastolic diameter *z*-score was 10.8. All patients received HF medications. Five patients (83%) showed normalization of LV function at a median age of 2.7 years. One patient progressed to end-stage HF requiring heart transplant. This case series identified a unique phenotype of HF with reduced ejection fraction at presentation that evolved to HF with recovered EF in over 80% of infant DCM cases with LOF *VCL* variants. These findings have prognostic implications for counseling and management of *VCL*-associated DCM and highlight a possible genetic basis for HF with recovered ejection fraction.

**Graphical abstract:**

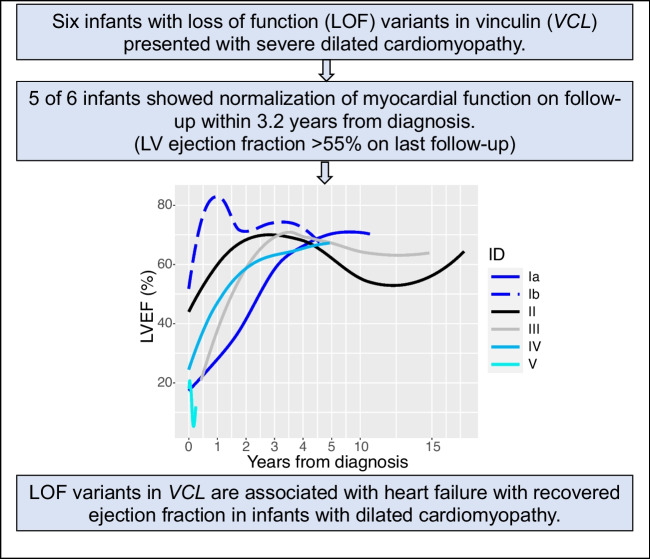

## Introduction

Dilated cardiomyopathy (DCM) is the most frequent cardiomyopathy in children, with 5-year transplant-free survival of 46%, and myocardial recovery in 20% [1, 2]. The etiology of pediatric DCM is complex and includes infectious, ischemic, nutritional, metabolic, oncologic and genetic causes. A genetic etiology is identified in less than 20% of cases, due in part to the uncertain association of several genes with cardiomyopathy [3, 4]. Vinculin is a gene with conflicting evidence for a role in DCM. Vinculin is a ubiquitously expressed protein encoded by the *VCL* gene with a larger splice isoform, metavinculin, exclusively expressed in cardiac and smooth muscle. Vinculin links the actin cytoskeleton to the cell membrane which is critical in force transmission and is needed to maintain cardiomyocyte function [5, 6]. Heterozygous *VCL* knockout mice develop DCM with abnormal adherens junctions and breakdown of the intercalated disc structure by 6 months of age [5]. In human studies, rare loss-of-function (LOF) variants in *VCL* were enriched in DCM patients compared to population controls (odds ratio 11.3–21.3), and often associated with infantile-onset disease [6–9]. However, there are also reports of reduced penetrance of putatively pathogenic variants. As a result, *VCL* variants are often classified as variants of uncertain significance (VUS) by most clinical laboratories leading to challenges in genetic counselling and unclear implications for families [8]. To clarify the clinical consequences of these variants, we undertook a genotype-phenotype association study. Here, we describe a unique phenotype of heart failure with reduced ejection fraction (HFrEF) that evolves to HF with recovered ejection fraction (HFrecEF) in DCM patients with *VCL* LOF variants*.*

## Methods

Patients with DCM enrolled in the Heart Centre Biobank Registry followed at a tertiary care pediatric cardiomyopathy referral center who harbored a non-benign LOF variant in *VCL* on whole genome sequencing (confirmed on clinical genetic testing) were included. The sequencing details and workflows have been previously published [10]. LOF variants (frameshift, splicing, nonsense) that were classified as pathogenic, likely pathogenic, or VUS using American College of Medical Genetics criteria were included [11]. Allele frequency was determined in Genome Aggregation Database (gnomAD) v2.1.1. Clinical and echocardiographic data were ascertained from patient medical records. All patients underwent an extensive clinical work-up to exclude secondary causes of cardiomyopathy including viral and metabolic screening, inflammatory biomarker assessment, and echocardiographic assessment for coronary abnormalities. Results were negative in all patients. Echocardiographic data was acquired serially from diagnosis until last follow-up and included left ventricular ejection fraction (LVEF) and LV end-diastolic diameter (LVEDD) indexed to body surface area using Boston *z*-scores. LVEF >55% was defined as normal ventricular function, and LVEDD *z*-score <2 as normal LV size. HFrecEF was defined as normalization of LV systolic function (LVEF ≥55%). The study was approved by the institutional Research Ethics Board and families provided written informed consent.

## Results

**Fig. 1:**
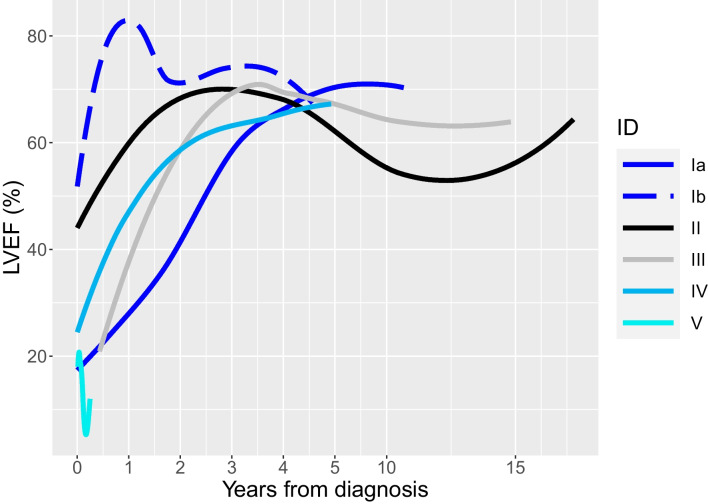
Left ventricular ejection fraction (LVEF) from diagnosis to last follow-up (*n*=84 echocardiograms from 6 patients). LVEF normalized in all patients except patient V who showed a progressive decline in LVEF

**Fig. 2:**
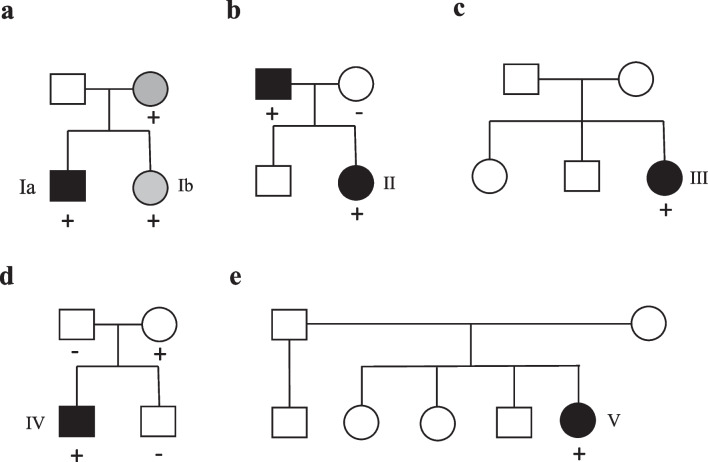
Family pedigrees of patients harboring *VCL* variants. **a** Patient Ia, Ib - *VCL*, c.1639C>T, (p.Arg547X); **b** patient II—*VCL*, c.3115C>T, (p.Gln1039X); **c** patient III—*VCL*, c.2949del, (p.Lys983fs); **d** patient IV - *VCL*, c.670_671insG, (p.Glu224fs); **e** patient V—*VCL*, c.313C>T, (p.Arg105X). Square = male, circle = female; white = unaffected, black = DCM, grey = mild phenotype (arrhythmias or mild cardiac dysfunction); + genotype-positive for *VCL* variant, − genotype-negative for *VCL* variant

The clinical, genetic findings and outcomes of 6 patients with DCM and a *VCL* LOF variant (from 5 families) are described (Tables 1 and 2). The median age at diagnosis was 2 months, median LVEF was 24%, and median LVEDD *z*-score was +10.8. All patients showed LV dilation and dysfunction on echocardiograms and LV or biventricular hypertrophy and *T* wave abnormalities on electrocardiogram. All patients harbored a *VCL* LOF variant that was either absent or very rare in gnomAD (minor allele frequency ≤0.0001). Variants were classified by the clinical laboratory as a VUS in 5 patients and likely pathogenic in one (patient IV). All five *VCL* variants were in coding regions in both the NM_014000.2 and NM_003373.3 transcripts. Four patients harbored additional VUS in other cardiomyopathy genes. All patients received an angiotensin-converting enzyme (ACE) inhibitor and a beta-blocker. Overall, 5 infants showed normalization of ventricular function during follow-up. Time from diagnosis to recovery ranged from 0.3–3.2 years (median age at recovery, 2.7 years) with median age at last follow-up of 11 years. One patient progressed to end-stage HF requiring a heart transplant in infancy. Figure 1 shows serial LVEF from diagnosis to last follow-up. Detailed case descriptions are provided below and family pedigrees are shown in Fig. 2.

**Table 1:** Baseline and follow-up clinical and echocardiographic characteristics and outcomes (*n*=6)

Family ID	Sex	Diagnosis	Age (years) at diagnosis	Age (years) at recovery of function/VAD	Time (years) to recovery/VAD	LVEF (%) at diagnosis	LVEDD *z*-score at diagnosis	HF medications	Inotropes	Last follow-up age (years)*	Last follow-up LVEF (%)*	Last follow-up LVEDD *z*-score*	HF medications at last follow-up	Outcome
Ia	M	DCM	0.47	3.7	3.2	11	8.5	Y	N	11.1	70	2.1	ACE-inhibitor (being weaned)	Recovered EF
Ib	F	Mild LV dysfunction	0.03	0.3	0.3	50	-1.8	Y	N	7.2	67	-1.9	Discontinued @ age 2yrs	Recovered EF
II	F	DCM, LVNC	0.05	1.0	1.0	47	N/A	Y	Y	17.3	65	0.9	ACE-inhibitor	Recovered EF (1.05y); relapsed (10.7y); recovered (14.6 y)
III	F	DCM	0.03	2.2	2.1	30	10.8	Y	N	14.8	65	1.6	Discontinued at 12yrs old	Recovered EF
IV	M	DCM	0.52	2.7	2.2	16	13.1	Y	N	8.4	65	3.4	ACE-inhibitor, β-blocker (being weaned)	Recovered EF
V	F	DCM	0.35	0.6	0.3	19	11.1	Y	Y	0.6	12	15.4	Not applicable	VAD (7mo); Transplanted (10mo)

*Last follow-up or before VAD

*DCM* dilated cardiomyopathy, *LVNC* left ventricular non-compaction, *LVEF* left ventricular ejection fraction, *LVEDD* LV end-diastolic diameter, *HF* heart failure, *VAD* ventricular assist device, *ACE-I* angiotensin-converting enzyme inhibitor, *β-blocker* beta-blocker, *y* years, *mo* months

**Table 2:** Genetic characteristics (*n*=6)

Family ID	Family history of cardiomyopathy	*VCL* variant identified	Variant type	gnomAD allele frequency	Clinvar annotation	ACMG classification	Zygosity	Inheritance	Other VUS
Ia	Yes (sibling)	*VCL*, c.1639C>T, (p.Arg547X)	Truncating	0.00001	VUS	VUS	Heterozygous	Unaffected mother	*DSP*, c.943C>T (p.Arg315Cys); *TTN*, c.41709G>T (p.Trp13903Cys)
Ib	Yes (sibling)	*VCL* c.1639C>T (p.Arg547X)	Truncating	0.00001	VUS	VUS	Heterozygous	Unaffected mother	*DSP*, c.943C>T (p.Arg315Cys)
II	Yes (father)	*VCL*, c.3115C>T, (p.Gln1039X)	Truncating	0	VUS	VUS	Heterozygous	Affected father	None
III	No	*VCL*, c.2949del (p.Lys983fs)	Frameshift	0	VUS	VUS	Heterozygous	Unknown	*MYBPC3*, c.3628-41_3628-17del; *LDB3* c.2078C>A (p.Thr693Asn)
IV	No	*VCL*, c.670_671insG, (p.Glu224fs)	Frameshift	0	Likely pathogenic	VUS	Heterozygous	Unaffected mother	*PKP2,* c.725C>T (p.Thr242Met)
V	No	*VCL*, c.313C>T, (p.Arg105X)	Truncating	0.00001	VUS	VUS	Heterozygous	Unknown	None

*gnomAD* Genome Aggregation Database, *ACMG* American College of Medical Genetics, *VUS* variant of uncertain significance

Patient Ia presented at 5.5 months of age with poor feeding and breathlessness. Echocardiogram revealed a severely dilated LV and severely reduced LV systolic function in addition to mechanical dyssynchrony. Metabolic and infectious work-up was non-contributory. Patient was treated with captopril, carvedilol, furosemide, and received supplemental enteral feeds until age 3 years. Echocardiography showed normal LVEF at age 3.7 years. He was weaned off carvedilol and furosemide and is currently weaning off perindopril with normal LV function at last follow-up at 11 years of age. Genetic testing revealed a rare LOF variant in *VCL* c.1639C>T (p.Arg547X) that has not been previously reported. It was initially classified as likely pathogenic and subsequently reclassified as a VUS by the clinical laboratory. Two VUS in *DSP* and *TTN* were also identified that were relatively frequent in gnomAD. The *VCL* and *TTN *variants were inherited from the mother who had a normal echocardiogram but had arrhythmias on Holter monitoring at last follow-up, and the DSP variant was inherited from an unaffected father. The DSP variant has been reported previously in the context of a second pathogenic variant and was not considered disease-causing. Patient Ib, sibling of patient Ia, harbored the *VCL* and *DSP* variants, and had mildly reduced LVEF of 50% on newborn echocardiogram in the context of a normal pregnancy and delivery. Patient was started on captopril with normalization of cardiac function by three months. Captopril was discontinued at two years of age with normal heart function at last follow-up at age 7.2 years.

Patient II had a relapsing course. She was diagnosed at 6 months with DCM with LV non-compaction, a small secundum atrial septal defect that closed spontaneously by age 4 years, and mild tricuspid valve dysplasia with moderate tricuspid regurgitation, unlikely to account for LV dysfunction. She was treated with an ACE-inhibitor and beta-blocker. LV function normalized by age 1 year and medications were discontinued. The LVEF decreased again at age 10.7 years, and she was restarted on an ACE-inhibitor. She again showed normalization of LV function by age 14.6 years that was sustained at last follow-up. The *VCL* variant was inherited from her affected father.

Patient III was diagnosed at age 3 months with severe DCM and was treated with an ACE-inhibitor and beta-blocker. She showed normal LV function by age 2.2 years and was weaned off all HF medications by age 12 years with normal LV function at last follow-up at 14.8 years.

Patient IV was diagnosed at age 6 months with DCM with severe LV dilation and dysfunction and was treated with an ACE-inhibitor and beta-blocker. LV function normalized by age 2.7 years with normal function at last follow-up at age 8.4 years on weaning HF medications. The *VCL* variant was inherited from her unaffected mother.

Patient V who was diagnosed with severe DCM at age 4 months progressed to decompensated HF requiring a LV assist device at age 7 months and a heart transplant at age 10 months. She is alive and well at last follow-up.

## Discussion

We describe a unique cardiac phenotype in patients with *VCL* LOF variants characterized by infantile-onset of severe DCM and evolution from HFrEF to HFrecEF. Patients presented in infancy but a majority (83%) showed normalization of LV function by 3 years of age. HFrecEF is recognized as a distinct type of HF often secondary to ischemia, toxins, or myocarditis [12]. To date, studies have not identified genetic factors associated with HFrecEF. Our study suggests that HFrecEF may occur in a subset of DCM patients with a distinct underlying genetic etiology.

Previous studies have identified younger age at diagnosis, lower LVEDD *z*-score and female sex as predictors of recovery in DCM, with only 13% of patients with a *z*-score > +6.29 showing recovery of function [13, 14]. In our cohort, only patient Ib had a mild phenotype because they were screened from birth and started on HF medications early which may have prevented disease progression. Four patients showed normalization of LV function despite severe LV dilation and dysfunction at presentation; only one patient progressed to end-stage HF requiring transplantation. Overall, the 83% frequency of recovery is much higher than the 20–30% recovery reported in previous studies of DCM [2]. While the presence of VUS in other cardiomyopathy genes may have contributed to the phenotype in some patients, an assessment for gene-gene interactions is beyond the scope of this study.

Of note, 3 patients were weaned off all HF medications of which only one patient relapsed requiring re-initiation of an ACE-inhibitor with subsequent normalization of LV function. The other two remained on single or dual HF medications that are being weaned at this time. Longer follow-up will help ascertain if recovery is permanent which in turn will help guide decision-making regarding how long to continue HF medications in those who show recovery. The mechanism of recovery of cardiac function is unclear. Vinculin has been implicated in stabilizing E-cadherin on the cell surface by interacting with β-catenin [15, 16]. It is possible that vinculin haploinsufficiency may be more disruptive in infancy when E-cadherin is still immature. Once vinculin co-localizes into the intercalated disk as patient matures, the consequences of its insufficiency may become less pronounced and may result in regression of the phenotype.

The serial evaluation is a strength of our study. It allowed us to capture the evolving cardiomyopathy phenotype unlike previous cross-sectional studies that may have missed an earlier diagnosis of DCM in recovered older patients resulting in the perception of reduced penetrance of VCL variants. Another strength was the use of comprehensive genomic sequencing that allowed us to identify *VCL* variants which may not be routinely included in all clinical test panels, as well as exclude other pathogenic variants. Similar to previous reports that identified a LOF *VCL* inherited from an unaffected parent in 5 of 9 families where segregation data was available, the *VCL* variant was inherited from an unaffected parent in 2 of our families [9]. This may imply either recovered DCM in the parent, or that *VCL* variants may be genetic modifiers in the context of other genetic or environmental factors [8].We did not identify fetal or postnatal insults or other pathogenic genetic variants that could contribute to cardiomyopathy. *VCL* LOF variants therefore appeared more likely to be causal than contributory in our case series.

In summary, we identified a strong association of *VCL* LOF variants with severe infantile-onset DCM with a high frequency of sustained recovery of cardiac function on follow-up. Consequently, the classification of such variants as VUS may not adequately capture their clinical implications. Our findings have important prognostic implications for counseling regarding the potential for spontaneous recovery in patients with *VCL*-associated DCM. It also highlights that HFrecEF may have a genetic basis in some cases. Further studies are needed to determine the long-term outcome of patients with *VCL*-associated DCM with recovered EF.

## Data Availability

Sequencing data are deposited in the European Genome-Phenome Archive (EGA) under accession EGAS00001004929, and are available for download upon approval by the Data Access Committee. Additional data generated or analyzed during this study are available from the corresponding author on reasonable request.

## Abbreviations

ACE: Angiotensin-converting enzyme
DCM: Dilated cardiomyopathy
gnomAD: Genome Aggregation Database
HF: Heart failure
HFrEF: Heart failure with reduced ejection fraction
HFrecEF: Heart failure with recovered ejection fraction
LOF: Loss-of-function
LVEDD: Left ventricular end-diastolic diameter
LVEF: Left ventricular ejection fraction
VUS: Variant of uncertain significance

## Declarations

### Animal Studies

No animal studies were carried out by the authors for this article.
